# Benign Choledochal Cyst Management in Adults: General Surgeons' Perspective in a Non-Tertiary Centre

**DOI:** 10.7759/cureus.89161

**Published:** 2025-07-31

**Authors:** Md M Buksh, Ahmad Al Samaraee

**Affiliations:** 1 General Surgery, Ashford and St. Peter's Hospitals NHS Foundation Trust, Chertsey, GBR

**Keywords:** bile duct, biliary cyst, cholangiocarcinoma, choledochal cyst, congenital biliary dilatations

## Abstract

Background: Choledochal cysts (ChDCs) in adults are rare and mostly benign. Their management involves having either extensive surgery in tertiary centres or conservative long-term surveillance in general hospitals. However, there is a gap in the literature regarding the ideal management approach to adopt in practice. This study explored our experience in this context.

Methods and analysis: This three-year retrospective study was conducted in a district general hospital in the West. The database of magnetic resonance scans of the pancreaticobiliary territory in adults over the study period was reviewed. Patients diagnosed with choledochal cysts through these scans were included in the final analysis. Descriptive statistics were used to analyse the collected quantitative data.

Results: Two thousand thirteen relevant scans were reviewed. Twenty-nine (1.44%) scans showed various types of ChDCs, where the patients' mean age was around 48 years, the female to male ratio was (2.6:1), 70% were Caucasian and around 51% had Type I ChDC. Abdominal pain was the main associated symptom in around half of them. None of the patients had objective evidence of malignancy that would warrant ChDC excision, while one-third had gallstones that necessitated cholecystectomy. All patients had conservative management and were still under surveillance at the end of this study. Neither mortality nor serious morbidity was reported.

Conclusions: Our findings are consistent with other studies, although we found higher incidence of ChDCs in Caucasians perhaps due to geographical reasons. With the current gap in the literature, we encourage other units to publish their similar experiences. This can help in standardisation of the management protocols of this rare pathology in adults.

## Introduction

Bile duct cysts or choledochal cysts (ChDCs) are dilatations of any part of the extrahepatic and/or intrahepatic biliary tree. In 1977, Todani et al. published a landmark paper that classified ChDCs into five types [1]. This classification is still being widely applied in practice; nevertheless, a sixth type has been increasingly reported in the literature over the recent years [2,3].

ChDCs are more common in Asian females, where they are typically presented in infancy and childhood [4]. The presentation however can be delayed until adulthood in nearly 20% of the cases [4]. The prevalence in the West varies between 1:100000-150000, while it is much higher in Asia where it is reported to be around 1:1000 live births in Japan [4-7]. Overall, ChDCs are rare, with an incidence of 0.1% in adult patients who undergo endoscopic retrograde cholangio-pancreaticography (ERCP) [8].

The pathophysiology that leads to the development of ChDCs is still not very well understood. It has been suggested that ChDCs are associated with congenital anomalies of the pancreaticobiliary junction (APBJ) or the presence of a small number of ganglionic cells in the wall of the bile ducts due to congenital factors [5,9,10].

The process of diagnosing ChDCs in adults is often challenging, since most patients report a long history of nonspecific upper abdominal pains [9]. However, they can occasionally be presented with jaundice, abdominal mass or pancreatitis [9].

Moreover, there are no specific laboratory tests that can help in establishing the diagnosis [9,11]. Therefore, diagnosis of ChDCs is usually based on radiological findings, keeping in mind that ChDCs are not uncommonly reported to be an incidental finding during various radiological investigations in adults [2].

ChDCs can carry long-term risks of recurrent morbidities and even a potential for mortality through their well-recognized complications like recurrent cholangitis, liver abscess, pancreatitis, biliary cirrhosis, biliary strictures, cyst rupture, portal hypertension and stone formation in the biliary system and pancreatic duct [12].

Most importantly, ChDCs in adults can be associated with a non-negligible long-term risk of developing cholangiocarcinoma. In one large systematic review/meta-analysis of 2904 adult patients who had ChDCs, this serious risk was reported in 10.7% of the included patients in the study [13]. It should also be kept in mind that the prognosis of patients who develop malignancy is poor, with a five-year survival rate of around 5% [14]. 

Due to this associated malignancy risk, there is no role for cyst drainage only in adults [2]. Depending on the type of cyst and whether it is associated with APBJ, the main surgical principle in adults is based on performing total excision of the ChDC, with restoration of the bile flow where applicable [2,9,11]. The alternative approach to surgery is the application of conservative management through long-term clinical and radiological surveillance. These surveillance protocols mostly take place at general hospitals under the care of general surgeons, but only through close liaison with the regional hepatobiliary multidisciplinary teams (HPB-MDT). In addition, the patients usually have a significant role in the setting of their management strategy (i.e. surgery versus long-term annual clinical and radiological surveillance). However, there is a current gap in the available literature about the best approach to adopt in practice (i.e. surgery vs. surveillance only) [13,14].

Magnetic resonance (MR) scans of the pancreatico-biliary territory have been well reported to be the radiological modality of choice in establishing the diagnosis and surveillance of various biliary tree pathologies like ChDCs, with a reported accuracy of >90% in this context [15,16]. The authors have implemented this well-recognized evidence in conducting this three-year retrospective study, which has looked into our District General Hospital (DGH) experience in conservative management of benign ChDCs in adults and its associated challenges.

## Materials and methods

This retrospective study was conducted at a DGH setting in the United Kingdom (UK). It had looked into our experience in conservative management of benign ChDCs in adults between April 2020 and April 2023. The electronic database of all pancreatico-biliary related MR scans reports over the study period (regardless of the MR scans' indication) was reviewed to identify the relevant cases for this study. All adult patients with an established diagnosis of any type of ChDCs through these MR scans were included in the final analysis. The ChDC MR feature of the included cases is a fluid-filled cystic structure involving any part of the biliary tree, which is hypointense on T1 images and hyperintense on T2 images. We excluded any scans that showed bile duct dilatation only without these radiological features. We have also excluded paediatric cases. The electronic clinical records of the included patients were reviewed and carefully matched against the regional HPB-MDT data. Each included patient has a record in our regional HPB-MDT data, which includes the patient's management plans and their surveillance outcome. The retrieved final data were presented on a spreadsheet according to certain parameters. These parameters included the patient`s age, gender, ethnicity, mode of presentation/symptoms, number and duration of admissions to DGH, MR scan findings, type of ChDC, associated APBJ, associated gallstones disease, whether they had cholecystectomy, whether they had surgery for ChDCs, surveillance outcomes, and mortality. Descriptive statistics were used to analyse the collected quantitative data. This study does not contain information that might have any potential for patient identification. 

## Results

The initial cohort was composed of 2013 various pancreatico-biliary MR scans that were performed in adults over the study period. These included 1915 magnetic resonance cholangiopancreatography (MRCP), 96 MR pancreas with contrast, and two secretin-enhanced MRCP (S-MRCP) scans.

**Figure 1:**
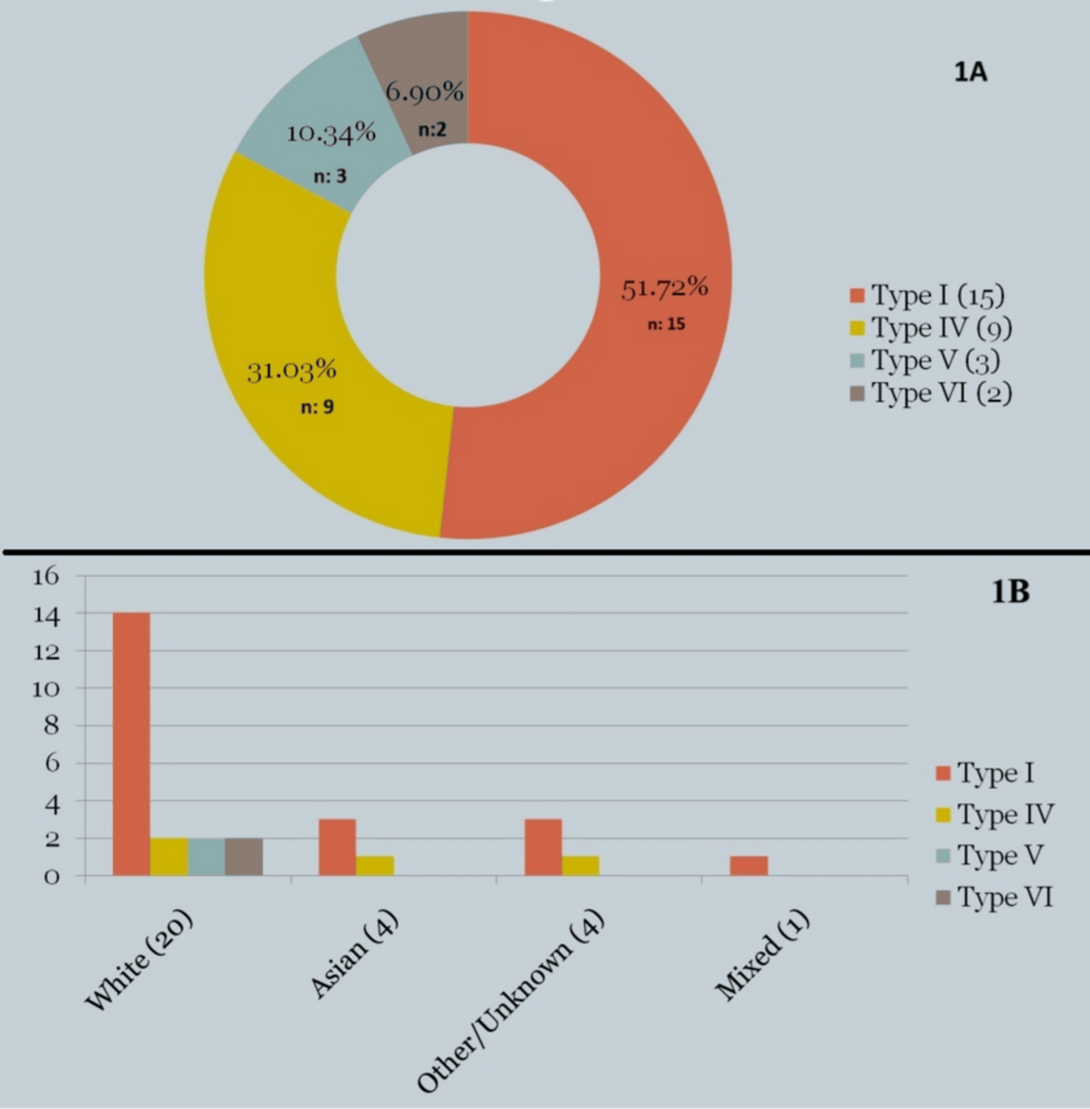
Correlation between Choledochal Cysts and Ethnicity 1A (Types of ChDCs): This shows the frequency of the types of ChDCs. 1B: (Ethnicity & ChDCs type): This shows the correlation between type of ChDCs and patient ethnicity. ChDCs: Choledochal Cysts

Twenty-nine of 2013 (1.44%) scans met the inclusion criteria by reporting various types of ChDCs. These 29 cases were included in the final analysis. The mean age of the 29 patients was around 48 years (range: 24-77), with a female-to-male ratio of 2.6:1. 15/29 (51.7%) of the patients had Type I ChDC, while types II and III were not reported in any patient (Figure 1). On the other hand, three of 29 patients (10.34%) with Type I ChDC had associated APBJ in the form of pancreatic divisum. Seventy percent of the patients were Caucasians while 13.8% of them were Asians. The remaining patients were of other ethnicities (Figure 1).

Non-specific but significant upper abdominal pain was the main presenting symptom in more than half of the cases (Table 1). These needed a total of 33 admissions to DGH purely for pain control. On the other hand, the average duration of admissions was 72 hours. Throughout their frequent admissions, and in order to identify a clear cause of their difficult-to-control abdominal pains, these 14 patients were thoroughly and repeatedly investigated by MR scans, abdominal ultrasound scans, computed tomography (CT) scans of the abdomen and endoscopy. However, the repeated tests completely failed to show other pathologies (rather than ChDCs) that could potentially explain the patients' troublesome pain symptoms.

**Table 1:** Patients' Modes of Presentation/Symptoms Summary of the modes of presentation of the 29 patients with choledochal cysts

Presentation	Number of patients	Percentage
Upper abdominal pain	16	55.2%
Incidental findings during radiological investigations	5	17.3%
Abnormal liver function tests	4	13.8 %
Nausea and Vomiting	2	6.9%
Pancreatitis	1	3.4%
Incidental intraoperative finding	1	3.4%
	Total: 29 patients	

Other modes of presentation were less commonly reported, like abnormal liver function tests (LFTs), nausea and vomiting, and pancreatitis. In addition, around one-fifth of the cases were found incidentally (Table 1). It is worth mentioning here that five of the incidental cases did not have any symptoms related to their ChDCs. The sixth case however was found incidentally during an elective laparoscopic cholecystectomy. All six patients had MRCP scans that confirmed the presence of Type I ChDC.

All of the 29 patients who were included in the study did not have significant co-morbidities. The American Society of Anesthesiologists (ASA) grade was one for about 70% of them, while the rest had ASA grade of two. Therefore, they were all considered clinically suitable to have complex surgery. Despite this, all of them preferred to have conservative management only through clinical and radiological annual surveillance.

About one-third of the 29 patients had associated symptomatic gallstone disease requiring cholecystectomy at some stage over the study period. None of the 29 patients had excision of the ChDC or objective evidence of malignancy. Neither significant morbidity nor mortality was reported.

The management and annual surveillance plans were decided at the regional HPB-MDT meeting level. All of the 29 patients were still under annual clinical and radiological surveillance at the end of this study.

## Discussion

ChDCs in adults are rare and mostly benign (Table 2). However, they can be associated with malignancy in 10.7% of the cases [13]. In current practice, the management options are surgery or long-term surveillance.

**Table 2:** Classification and Prevalence of Choledochal Cysts Anatomical Description of Types I-VI Choledochal Cysts [2]

Category	Description	Prevalence
Type I	Dilatation of the extrahepatic bile duct	50-85% of the cases
Type II	Diverticulum of the extrahepatic bile duct	2% of the cases
Type III	Dilatation of the intra-duodenal portion of the common bile duct (choledococele)	1.4% - 4.5% of the cases
Type IV	Type IVA: multiple cystic dilatations of the intrahepatic and extrahepatic bile ducts; Type IVB: multiple cystic dilatations of the extrahepatic bile duct only	15% - 35% of the cases
Type V	Cystic dilatation of the intrahepatic bile ducts (Caroli's disease)	20% of the cases
Type IV*	Cystic dilatation of the cystic duct *(Not included in Todani's Classification)	Extremely rare

Total excision of the ChDC and restoration of the bile flow is the main surgical principle in this perspective (Table 3). Generally, the surgical options can be applied through open, minimally invasive or combined approaches, depending on the type of ChDC, the local protocols (Table 4) and the available surgical expertise and resources [2,9,12]. After surgery, the patients should also be kept under long-term surveillance protocols due to the persistent long-term risk of malignancy even after surgical excision of the cysts [2,12]. One of the commonest surgical interventions in adults is Roux-en-Y Hepaticojejunostomy (RYHJ) for Type I and Type IV ChDCs. However, this operation can be associated with significant morbidity in the short term like bile leak, and long-term complications like intrahepatic cholelithiasis, recurrent cholangitis, liver abscess, pancreatitis, and anastomotic stricture [9,17]. The risk of developing anastomotic stricture is reported to be around 12.5% in two years and 40% in five years following RYHJ [18]. One should also keep in mind that the failure rate of surgical revision for anastomotic strictures complicating RYHJ can be as high as 50% [19]. In recent years, endoscopic and percutaneous interventions have become the forefront in managing this type of stricture through balloon dilatation and repeated stenting [20]. However, this approach is not free of associated risks, in addition to its non-negligible burden on the patients' lifestyle and the health services [20].

**Table 3:** Surgical Principles in Managing Choledochal Cysts in Adults Recommended general surgical principles in managing choledochal cysts (ChDCs) in adults. The choice of the type of surgical intervention would depend on the type of the choledochal cyst and whether it is associated with anomalies of the pancreaticobiliary junction [2,9,11]. RYHJ: Roux-en-Y Hepaticojejunostomy

ChDC Type	Recommended Surgical Principles
Type I	Total excision of the extrahepatic biliary tree (including the ChDC), with restoration of bile flow by RYHJ.
Type II	Simple excision of the cyst or diverticulectomy
Type III	For small cyst: Endoscopic sphincterotomy and cyst de-roofing, with biopsy of the ChDC mucosa; For large cyst: Transduodenal excision of the cyst, with marsupialization into the duododnal mucosa
Type IV	Total excision of the extrahepatic biliary tree (including the ChDC), with restoration of bile flow by RYHJ.
Type V	Segmental hepatic resection for localized or unilobular disease; Liver transplant for diffuse disease
Type VI	Standard cholecystectomy for isolated ChDC with narrow junction between the cyst duct and the bile duct; Total excision of the extrahepatic biliary tree and RYHJ for wide junction between the cystic duct and the bile duct or when it is associated with Type I ChDC (i.e. when Type I & Type VI ChDCs exist together)

**Table 4:** Summary of Our Local Protocol in the Context of Managing Benign Choledochal Cysts in Adults. ERCP: endoscopic retrograde cholangio-pancreaticography, EUS: endoscopic ultrasound scan, HPB MDT: hepatobiliary multidisciplinary team

Local Protocol
Decision making always takes place at the regional HPB MDT level (including the patients who are on long-term follow-up).
Patients have a role in the setting of their management strategy [surgery versus long-term annual clinical/radiological (MR) follow-up].
EUS is indicated in patients who can't have MR scans, or have ongoing pains without radiological evidence of biliary system stones/sludge.
ERCP for associated bile duct stones.
Laparoscopic cholecystectomy where applicable.

Therefore, the decision to go through these types of extensive surgical and endoscopic interventions is unsurprisingly a big step to take by the patients and surgeons, particularly in the younger group of patients who don`t have significant symptoms or any objective evidence of malignancy in association with their ChDCs.

As a result, the application of an alternative conservative approach in managing benign ChDCs in adults has been adopted in practice. This approach involves close monitoring through long-term clinical and radiological surveillance protocols. These are mostly performed in DGH settings, but only through decision making at the regional HPB-MDT level, and not forgetting the vital role of the patients in the setting of their management strategy (i.e. surgery vs. conservative management with long-term surveillance) [21]. It should be kept in mind, however, that patients who are under surveillance only plans must be strongly advised to seek urgent clinical review if they develop any red flag worrying symptoms like abdominal pain, jaundice, anorexia, nausea, vomiting and unexplained weight loss.

Due to its high accuracy in detecting biliary and pancreatic tree pathologies when compared to other tests, MRCP is currently considered the radiological investigation of choice in annual surveillance protocols for ChDCs in adults, especially since it does not involve radiation or contrast use [9,15,16]. MR signs that would suggest malignant changes in ChDCs are irregular thickening of the cyst wall, mass formation and the presence of papillary nodules [22]. However, MRCP has some limitation in this context due to its lower efficiency in detecting minor ductal abnormalities or small Type III ChDCs, in addition to the standard contraindications to have MR scans in general [15,16]. In these scenarios, the alternative efficient investigation would be the use of endoscopic ultrasound scan (EUS) to be performed by experienced hands [23,24]. 

Although all adult patients with ChDCs have a lifelong risk of developing cholangiocarcinoma, there are particular groups of them who can have a higher malignancy risk. Therefore, they should be subjected to more intense lifelong clinical and radiological surveillance. These are the ones with types I and IV ChDCs, the presence of associated APBJ, the older age group and in those who had previously incomplete surgical excision of their ChDCs [4,9,25].

Shimotake et al. had conducted an interesting study in a group of paediatric patients who had surgery for APBJ [25]. The study showed that those patients had multistep genetic mutational events that can be possibly associated with a higher risk of developing cholangiocarcinoma [25]. However, this study has not been replicated in adults as far as we know. It is worth mentioning here that the serum tumour marker cancer antigen 19-9 (CA19-9) is highly expressed in the choledochal epithelium; hence, its serum levels can be raised in various biliary tree malformations and cholangiocarcinoma. Still, this is not considered to be a pathognomonic test in diagnosing and surveillance of biliary cysts [9,11].

Currently, there is no available strong evidence that has looked into the context of long-term conservative management and surveillance of benign ChDCs in adults as an alternative option to surgical excision. This is not surprising due to the rarity of this condition. According to the Japanese guidelines, immediate surgery should be offered to juvenile patients with ChDCs due to the long-term risk of developing malignancy; however, there are no clear evidence-based recommendations that can be efficiently applied in adults [26].

Therefore, most centres worldwide follow their own set of local or regional protocols that are based on their practical and cumulative experiences in managing benign ChDCs in adults, in addition to their institutional audit of practice and research activities [4,6,12-14,17].

Our retrospective study results generally match other published works from the aspects of age and gender distribution, the more common types of ChDCs, modes of presentation and the associated gallstones disease rates [4,27]. However, we have found that ChDCs are more common in adult patients of Caucasian ethnicity. This is an interesting finding since ChDCs are more commonly reported in Asians [2,9]. It should be kept in mind however that this study was conducted in a predominantly Caucasian community in the West.

Limitations of this study

This retrospective study was limited by the small number of cases that were included in the final analysis. This is not surprising due to the rarity of the explored condition. Small sample size studies are generally considered to be less reliable in their statistical outcomes, since they usually tend to have a very wide confidence interval. Hence, the power of the study would be unsurprisingly low [28,29]. That is in addition to the limitations of retrospective studies in general, since they are typically bound to have missing data that is combined with selection and recall biases. Nevertheless, low-grade evidence can still provide valuable data and identify the practical challenges of applying evidence to real practice [30]. This study presents a DGH experience in managing a rare pathology, where the authors have backed up their analysis with the available evidence. It should also be kept in mind that the related published evidence in this context is generally sparse and statistically weak due to the rarity of the condition.

## Conclusions

Choledochal cysts in adults are rare and mostly benign, but they can be associated with long-term risk of developing cholangiocarcinoma in a group of patients. The choice of having conservative management through long-term clinical and radiological surveillance versus excision of the cyst through extensive surgery remains a dilemma, especially with the current gap in the literature in this context. Our findings are consistent with the other limited published studies, although we have found higher incidence of ChDCs in Caucasians. This can perhaps be explained by the study's geographical location in the West.

The study has also demonstrated the associated significant challenges in managing these patients, particularly those who suffer from persistent or recurrent attacks of abdominal pains. This can lead to frequent admissions to district general hospitals under the care of the general surgeons, where repeating various radiological and endoscopic investigations are not usually required in nearly half of the patients. In addition, long-standing surveillance programs are usually needed in these patients because of the non-negligible risk of developing malignancy in the long term. One should also keep in mind that nearly one-third of the patients can have associated gallstone disease that would generally necessitate cholecystectomy at a DGH level. All this can potentially be associated with a non-negligible burden on the patients' lifestyle and the health providers.

We believe that this study`s outcome is relevant to all general surgeons who have their practice based in non-tertiary centres, since it reflects real-life experiences from the perspective of non-surgical management and surveillance activities of an uncommon surgical pathology. As far as we know, this is the largest study that has looked into the management of benign ChDCs in adults at a district general hospital level in our region. Due to the rarity of this condition and the paucity in the literature regarding its management challenges, we do encourage other units to publish their related data and experiences in this context. This can potentially improve the management of this complex condition in adults from the clinical and cost-effectiveness viewpoints, and facilitate the implementation of standardized databases, guidelines, and algorithms in practice.
